# Hard X-ray operation of X-ray gas monitors at the European XFEL

**DOI:** 10.1107/S160057752400331X

**Published:** 2024-06-05

**Authors:** Theophilos Maltezopoulos, Frank Brinker, Florian Dietrich, Wolfgang Freund, Jan Grünert, Ulf Fini Jastrow, Naresh Kujala, Joakim Laksman, Jia Liu, Kai Tiedtke, Thomas Tschentscher

**Affiliations:** a European XFEL Holzkoppel 4 22869Schenefeld Germany; b Deutsches Elektronen-Synchrotron DESY Notkestrasse 85 22607Hamburg Germany; Paul Scherrer Institut, Switzerland

**Keywords:** free-electron lasers, photon diagnostics, hard X-rays, X-ray gas monitors, Huge Aperture MultiPlier

## Abstract

X-ray gas monitors (XGMs) are operated at the European XFEL for non-invasive single-shot pulse energy measurements and average beam-position monitoring. The underlying measurement principle is the photo-ionization of rare gas atoms at low gas pressures and the detection of the photo-ions and photo-electrons created. These are essential for tuning and sustaining self-amplified spontaneous emission (SASE) operation, machine radiation safety, and sorting single-shot experimental data according to pulse energy. In this paper, the first results from XGM operation at photon energies up to 30 keV are presented, which are far beyond the original specification of this device. Here, the Huge Aperture MultiPlier (HAMP) is used for single-shot pulse energy measurements since the standard X-ray gas monitor detectors (XGMDs) do not provide a sufficient signal-to-noise ratio, even at the highest operating gas pressures. A single-shot correlation coefficient of 0.98 is measured between consecutive XGMs operated with HAMP, which is as good as measuring with the standard XGMD detectors. An intra-train non-linearity of the HAMP signal is discovered, and operation parameters to mitigate this effect are studied. The upper repetition rate limit of HAMP operation at 2.25 MHz is also determined. Finally, the possibilities and limits for future XGM operation at photon energies up to 50 keV are discussed.

## Abstract 2

The operation of X-ray gas monitors at the European XFEL at hard photon energies up to 30 keV is described, and the possibilities and limitations for future operation up to 50 keV are discussed.

## 1. Introduction

Free-electron lasers (FELs) operated in self-amplified spontaneous emission mode (SASE) are currently the most intense femtosecond light sources in the hard X-ray wavelength regime. However, the statistical nature of the SASE process leads to intensity, temporal profile and spectral fluctuations for consecutive photon pulses (Saldin, 2000 ▸). Additionally, the electron acceleration introduces arrival-time fluctuations of the electron bunches, which leads to a temporal jitter in pump–probe experiments with respect to asynchronized external optical laser. Therefore, several single-shot diagnostic tools have been developed to measure the pulse energy (Richter *et al.*, 2003 ▸; Sorokin, 2004 ▸), temporal profile (Frühling *et al.*, 2009 ▸; Azima *et al.*, 2018 ▸), spectral distribution (Laksman *et al.*, 2019 ▸, 2022 ▸; Kujala *et al.*, 2020 ▸) and arrival time (Maltezopoulos *et al.*, 2008 ▸; Sato *et al.*, 2020 ▸) of the FEL pulses.

Gas monitor detectors (GMDs) (Richter *et al.*, 2003 ▸; Sorokin, 2004 ▸; Tiedtke *et al.*, 2008 ▸) were developed at the free-electron laser in Hamburg (FLASH) for the extreme ultraviolet wavelength range. Meanwhile, many facilities in the world use gas-based online intensity diagnostics (Moeller *et al.*, 2011 ▸; Kato *et al.*, 2012 ▸; Tiedtke *et al.*, 2014 ▸; Song *et al.*, 2019 ▸). The newer, so-called X-ray gas monitors (XGMs) were developed to extend the operation range into the hard X-ray photon energies up to 16 keV (Sorokin, 2011 ▸; Sorokin *et al.*, 2019 ▸). The measurement principle is based on the photo-ionization of rare gas atoms; therefore, it does not degrade during operation and is virtually transparent. The XGMs were calibrated at the Metrology Light Source of the Physikalisch-Technische Bundesanstalt (PTB) (Gottwald *et al.*, 2019 ▸). XGMs can measure individual femtosecond X-ray pulse energies non-invasively with an absolute measurement uncertainty of 7–10%. The average (over tens of seconds) beam-position measurement uncertainty is of the order of ±10 µm.

XGMs contain two X-ray gas monitor detectors (XGMDs) and two huge-aperture multiplier (HAMP) chambers, as described in detail by Sorokin *et al.* (2019 ▸) and Maltezopoulos *et al.* (2019 ▸). At XGMDs, the photo-ions and photo-electrons are extracted in opposite directions by an electric field. Photo-ions are detected with Faraday cups and low-pass filtered with an integration time constant of tens of seconds. This way, the average photon flux can be determined in absolute terms, but not the single-shot pulse energy. In addition, the photo-electron signal is coupled out of the vacuum chamber over a resistor/capacitor, is electronically amplified and digitized with an analog-to-digital converter (ADC). This signal is pulse resolved, but not absolutely calibrated like the photo-ion signal described above. In order to calibrate the single-shot data to the photo-ion signal, they are arithmetically averaged at the intrinsic time scale of the photo-ion measurement and thereafter cross-calibrated. HAMP is also based on photo-ionization. It was developed for very hard X-rays, where the photo-ionization cross-section is significantly reduced and the signal-to-noise ratio (SNR) of the XGMD single-shot signal becomes too low. In the HAMP chamber, a repeller plate separates the photo-electrons from the ions and accelerates the ions towards the multiplier. The multiplier converts the photo-ions into electrons and amplifies them in a cascade. The electron signal is coupled out of the vacuum chamber, then electronically amplified and finally digitized with an analog-to-digital converter. The single-shot HAMP data are, like the XGMD photo-electron signal, originally in arbitrary units and have to be cross-calibrated as described above. Additionally, at HAMP the calibration depends on the amplifier voltage and has to be repeated whenever the voltage is changed. We also observed at the start-up of the HAMP detector that the calibration slightly drifts by a few percent, which might be caused by warming of the detector. More details on the HAMP operation are reported for SwissFEL by Juranić *et al.* (2023 ▸).

At the European XFEL (EuXFEL), XGMs were installed and, since 2017, operated (Maltezopoulos *et al.*, 2019 ▸) continuously and reliably at different photon energies ranging from 300 eV up to 3 keV in SASE3 (soft X-ray beamline), and 5 keV up to 24 keV in SASE1 and SASE2 (hard X-ray beamlines). EuXFEL operates in burst mode, where the burst train repetition rate is 10 Hz. Such a train with a time window of 600 µs can be filled with up to 2700 individual pulses. The highest intra-pulse train repetition rate is 4.5 MHz. Details of the photon diagnostic setup can be found in Grünert *et al.* (2019 ▸). The EuXFEL facility with accelerator, photon transport system and experiments is explained by Tschentscher *et al.* (2017 ▸) and Decking *et al.* (2020 ▸).

An increasing photon energy puts higher demands on gas-based diagnostics, as the photo-ionization cross-section and often also the commonly achieved SASE power decrease. If multi-pulse trains are used, the average XGMD ion signal remains (with a slightly enhanced uncertainty) resolvable, but the single-shot XGMD electron signal drops below the detection limit above approximately 18 keV (depending also on the machine performance). Here, HAMP can still resolve the weak single pulses if the multiplier voltage is set accordingly. Future plans for EuXFEL include even higher photon energies, approaching the direction of 50 keV (Chen *et al.*, 2021 ▸). To date, research and development has been carried out up to 30 keV (Chen, 2022 ▸).

In this paper, we present XGM operation at 20 keV up to 30 keV photon energy. We start with XGM single-shot pulse energy correlations between two consecutive XGMs at 20 keV, where both have been operated with HAMP. Then, we describe an intra-train non-linearity we found for the HAMP signal and how we can mitigate this effect. Additionally, we present the upper repetition rate limit of HAMP operation. We then describe how we extrapolated the cross-sections and ion-mean-charges for xenon and krypton into significantly higher photon energies, compared them with literature values and give an example for operation at 30 keV. To conclude, we estimate the XGM resolution for future photon energies up to 50 keV and summarize our findings.

## 2. XGM operation with HAMP at 20 keV

At higher photon energies, there are two challenging effects for the operation of the XGM: the decrease in both the photo-ionization cross-sections and the average SASE pulse energies. By increasing the number of pulses per train, the absolutely calibrated average photo-ion based signal can be maintained around (sometimes slightly higher) the usual uncertainty of 7–10%. However, averaging does not help to improve the single-shot XGMD signal. We found that, above 18 keV, the XGMD single-pulse SNR becomes insufficient even at the highest operationally possible gas pressure of 1 × 10^−4^ mbar and with the gas of the highest cross-section (Xe or Kr). Thus, at higher photon energies the HAMP detector must be used.

**Figure 1:**
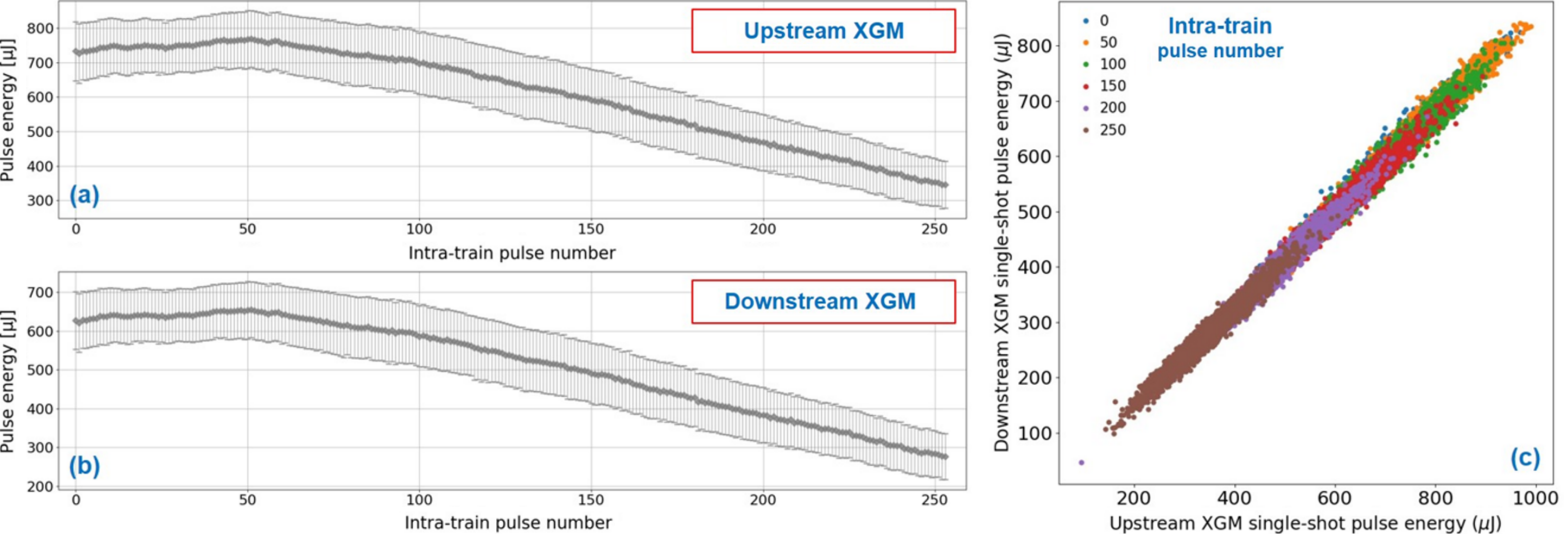
XGM operation at 20 keV with HAMP as the single-shot detector (SASE1 with 254 pulses per train, Xe gas target at both XGMs, both HAMP voltages at −1400 V, repeller at 6000 V, HAMP raw ADC peak around 1 mV but below 2 mV amplitude). Statistics over 2893 trains with the average and standard deviation (SASE jitter) for each pulse number in the train measured with the (*a*) upstream (XTD2) and (*b*) downstream (XTD9) XGMs. (*c*) Upstream XTD2 and downstream XTD9 single-shot linear correlation with a slope of 0.86 (beamline transmission) and correlation coefficient of 0.98 for a selection of some intra-train pulse numbers (color code).

An example for HAMP operation at 20 keV is shown in Fig. 1 ▸. Here, the SASE1 upstream XGM in tunnel XTD2 and the downstream XGM in tunnel XTD9 (SPB branch) were used with HAMP as a single-shot detector and Xe was used as the target gas. At both XGMs, the HAMP single-shot signal was calibrated to the integrational absolutely calibrated signal as described above. Figs. 1 ▸(*a*) and 1 ▸(*b*) show the variation in pulse energies along pulse trains for the upstream and downstream XGMs, respectively. Each data point is an average over 2893 trains for this position in the train. The error bars represent the standard deviation for the measurement run and illustrate the SASE jitter. Both XGMs measure the same pulse energy distribution over the train and show the same SASE pulse energy fluctuation of around 12–14%. Of course, the downstream XGM detects slightly lower pulse energies due to losses along the beamline. Note that EuXFEL can also produce homogeneous pulse energy distributions along the trains. This example was specifically selected to demonstrate the XGM performance. Fig. 1 ▸(*c*) shows single-shot pulse energy correlations between the upstream XTD2 (*x* axis) and downstream XTD9 (*y* axis) XGMs for a selection of several intra-train pulse numbers (see color code). All pulses show a linear correlation between the upstream and downstream XGMs with the same slope of 0.86, which corresponds to the transmission. The linear correlation coefficient is 0.98, which is as good as in the case of the usual XGMD detector at lower photon energies. Similar correlation coefficients have been found between other single-shot detectors at EuXFEL, like multi-channel plates, photo-electron spectrometers and diamond detectors relative to XGMs (not shown). A pulse with 600 µJ at 20 keV has 1.875 × 10^11^ photons. Using equation (1) (see below) with a temperature of 296 K, a pressure of 10^−2^ Pa, a length of 0.22 m and a cross-section of 0.0061 Mb (= 6.1 × 10^−25^ m^2^), this leads to *N* = 61600 ions per shot and to a statistical uncertainty of *N*^1/2^/*N* = 0.4%. Together, with the SNR of the ADC, this leads to a typical relative accuracy of a few percent.

Note that an XGM can be continuously operated with HAMP like in the case of the XGMD detector. These reliable single-shot measurements have been carried out with the HAMP signal around 1 mV but always below 2 mV and below 4.5 MHz repetition rate. The reason is that an intra-train non-linearity at HAMP for signals greater than 2 mV appeared together with overlapping HAMP peaks at 4.5 MHz, as shown in the next section.

## 3. Intra-train non-linearity and repetition rate limit of HAMP

In the correlation plot of Fig. 1 ▸(*c*), all pulses with different single-shot pulse energies and different intra-train pulse numbers are on the same correlation line with the same slope, as expected for linear detectors. This reliable and reproducible linear behavior is only found for HAMP signals below 2 mV.

**Figure 2:**
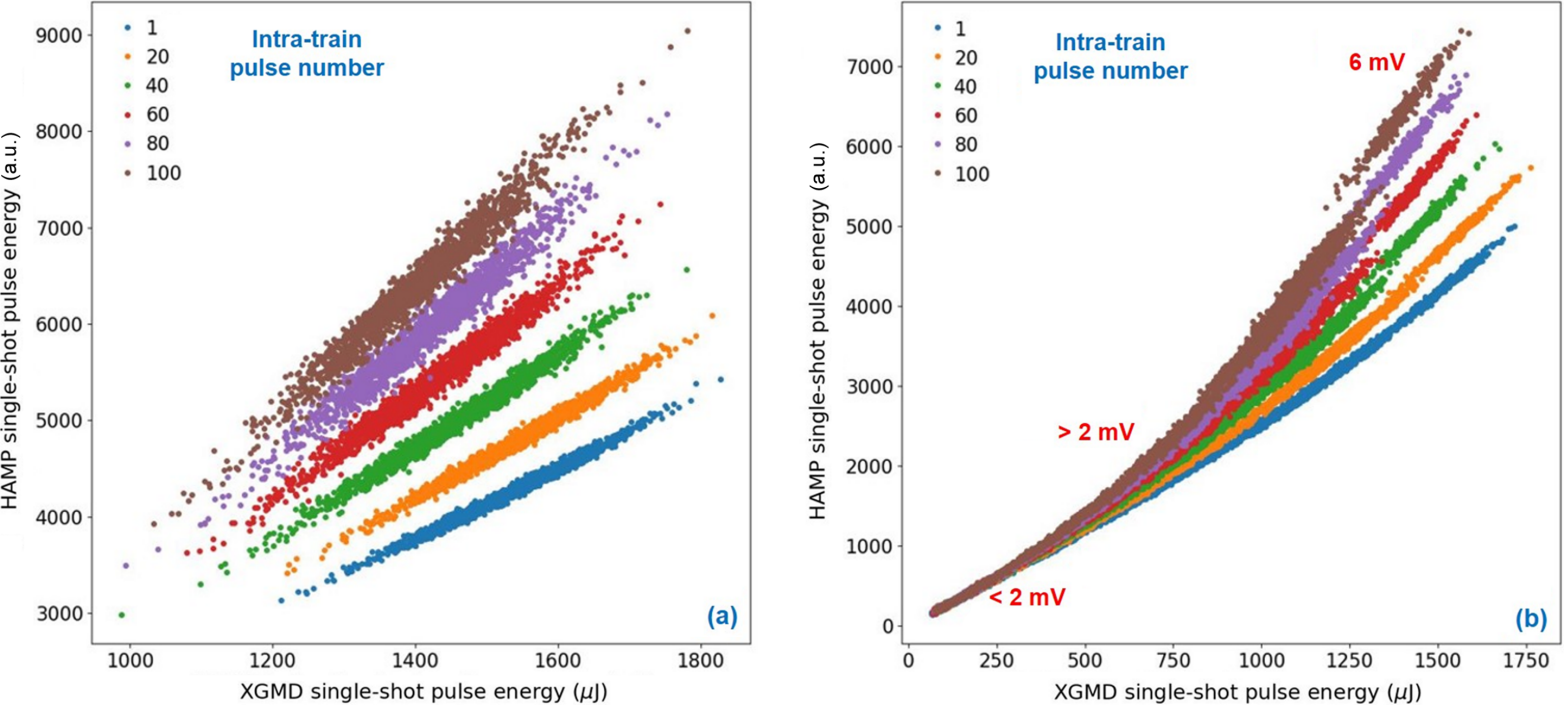
HAMP intra-train non-linearity (SASE3 at 780 eV with 120 pulses per train, Kr gas target, HAMP at −850 V, repeller at 6000 V). Intra-train non-linearity appears only for >10 pulses per train and only if the HAMP signal is >2 mV. In this case, HAMP voltage was used which leads to 6 mV peaks. The XGMD single-shot pulse energies were calibrated to microjoules whereas the HAMP single shots were kept in arbitrary units (because of the non-linearity). (*a*) Different correlation lines and slopes for pulses of different intra-train pulse numbers (color code). (*b*) Same settings as in (*a*), but with decreasing pulse energies during the DAQ run using the SASE3 gas attenuator. At low pulse energies, where the HAMP peaks are again <2 mV, the HAMP response is linear and the same for all intra-train pulse numbers.

A non-linear effect was found for HAMP signals above 2 mV. Fig. 2 ▸ shows correlations between XGMD and the HAMP detector of the XGM in the SASE3 Spectroscopy and Coherent Scattering (SCS) experimental hutch. This experiment was performed with soft X-rays of 780 eV, where at one single XGM the XGMD and the HAMP detectors could be used simultaneously with sufficient SNR. The HAMP detector was operated at a voltage of −850 V (repeller at 6000 V) which lead to HAMP peaks of around 6 mV. The XGMD single-shot values were calibrated to the absolutely calibrated photo-ion signal as described above, but the HAMP single-shot values were kept in arbitrary units (owing to the non-linearity).

Figs. 2 ▸(*a*) and 2 ▸(*b*) show single-shot pulse energy correlations between the horizontal SCS XGMD (*x* axis) and the vertical SCS HAMP (*y* axis) detectors of the same XGM for a selection of several intra-train pulse numbers (see color code). The plot in Fig. 2 ▸(*a*) shows different correlation lines for different intra-train pulse numbers. The later pulses in the train show a higher slope than those earlier in the train. The HAMP detector showed increased amplification after it had already amplified some pulses in the same train. Typically, this intra-train non-linearity was observed only for >10 pulses per train and only if the HAMP signal is >2 mV. This non-linearity was not observed with the XGMD detectors at FLASH or EuXFEL. For the plot in Fig. 2 ▸(*b*), the HAMP and XGMD settings were kept constant, but during the DAQ run the pulse energies were decreased continuously using the SASE3 gas attenuator (Dommach *et al.*, 2021 ▸). The transmission was decreased every 30 s from 100%, down to 90%, 80%,…, 10% and finally 5%. With decreasing pulse energies the HAMP and the XGMD peaks are reduced. When the HAMP peaks became smaller than 2 mV, the linearity returned and all pulses, independent of the pulse number in the train, were at the same line with the same slope.

**Figure 3:**
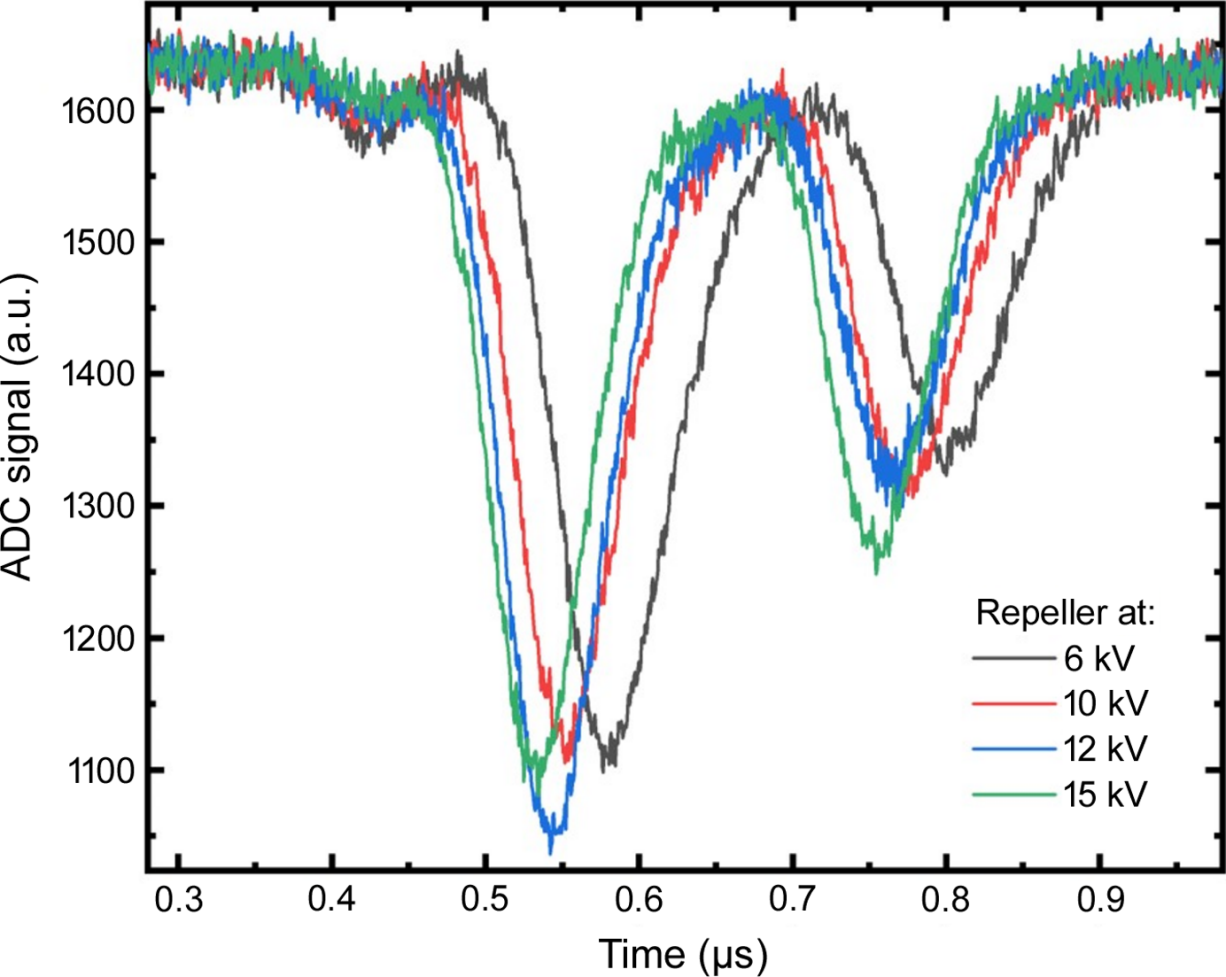
HAMP operation at 4.5 MHz repetition rate (2 pulses per train at 4.5 MHz or 222 ns, XTD1 XGM in SASE2 at 18 keV, Xe gas target, HAMP voltage at −1400 V). HAMP ADC traces at different repeller voltages as a function of time. HAMP peaks arrive at earlier times with increasing repeller voltage and the peak widths decrease, but consecutive peaks still overlap.

In addition to the non-linearity issue, which can be easily avoided by setting the HAMP to signals below 2 mV, next we discuss the repetition rate limit of the HAMP operation which we found to be 2.25 MHz. EuXFEL can deliver pulse repetition rates of up to 4.5 MHz, with lower rates of 2.25 MHz, 1.128 MHz, 564 kHz and so on. For comparison, HAMP data at 1.128 MHz are shown in an earlier publication (Maltezopoulos *et al.*, 2019 ▸). All repetition rates can be covered with the XGMD detector, but HAMP can only cover up to 2.25 MHz. At 4.5 MHz the HAMP peaks start to overlap and the zero level of the next pulse (and onwards) for the peak height or integral evaluation cannot be defined. Additionally, in longer pulse trains >30 pulses per train (not shown), a pile-up of the HAMP signal is observed. Note that HAMP operation between 2.25 MHz and 4.5 MHz could not be tested, thus the exact limit remains unknown. Fig. 3 ▸ shows raw single-train HAMP traces at 4.5 MHz with two pulses per train (pulse separation of 222 ns) at four different repeller voltages. These measurements have been carried out at SASE2 with 18 keV photon energy and Xe as the gas target. The single peaks move to earlier times with increasing repeller voltages, because the Xe photo-ions become faster and arrive earlier on the HAMP detector. As the repeller voltage increases, the first and second peak widths become 15% and 10% thinner, respectively, but unfortunately not enough to separate the peaks. We are planning experiments to use photo-electrons instead of photo-ions for HAMP and adapt the readout electronics accordingly, but currently HAMP cannot be used at 4.5 MHz repetition rate.

## 4. Extrapolation of cross-sections and ion-mean-charges for operation above 25 keV

To determine the average pulse energy from the averaged photo-ion signal, the XGM requires precise data tables for the ion-mean-charges and the photo-ionization cross-sections for the gases and photon energies used. These data tables were obtained at absolutely calibrated national metrology laboratories like the PTB in Berlin and often together with a bolometer for up to 25 keV (Kuehn, 2013 ▸, 2014 ▸). All XGMD measurement data, such as average photo-ion current, chamber pressure and temperature, but also all the data in these tables have a measurement uncertainty. All these uncertainties are used in an automatic error propagation to derive the final average pulse energy uncertainty. XGMDs were checked after many years of operation against bolometers (Saito *et al.*, 2010 ▸; Kato *et al.*, 2010 ▸; Grünert *et al.*, 2022 ▸) for different photon energies and gas types, and the measurements fit within the measurement uncertainty of both devices. Therefore, these data tables are very reliable and they are used on XGMDs at several facilities around the world. The following equations are used for XGMD signal evaluation: the number of ions per shot *N*_ion_ is given by the linear equation
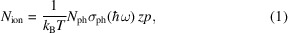
where *N*_ph_ is the number of photons per pulse, σ_ph_ is the photo-ionization cross-section, *z* is the XGMD detector length, *p* is the pressure, *k*_B_ is the Boltzmann constant and *T* is the temperature. The ion current *I*_ion_ is given by the linear equation
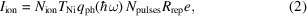
where *T*_Ni_ is the transmission of the Ni mesh in front of the Faraday cup (typically 0.8), *q*_ph_ is the ion-mean-charge, *N*_pulses_ is the number of pulses per train, *R*_rep_ is the train repetition rate (at EuXFEL it is 10 Hz) and *e* is the elementary charge.

There are future plans at EuXFEL for operation above 25 keV (Chen *et al.*, 2021 ▸; Chen, 2022 ▸). Since XGM is one of the main diagnostics at many FELs, it is necessary to extend its operation range above 25 keV. Obtaining new calibration data at synchrotron facilities is limited by the available photon flux, for example, the absolutely calibrated BAM PTB line B8 at BESSY II offers from 8 keV up to 60 keV about 10^7^ photons s^−1^ monochromatic light (Görner *et al.*, 2001 ▸). Taking equation (1) (calculated per second and not per pulse) at 29.3 keV with an Xe cross-section [taken from White (1934 ▸)] of 0.00195 Mb (= 1.95 × 10^−25^ m^2^) together with a detector length of 0.22 m, the maximum pressure of 1 × 10^−4^ mbar (= 10^−2^ Pa) and a temperature of 296 K, result in about 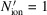 Xe ions s^−1^. Since we already have ions s^−1^, equation (2) can be simplified to *I*_ion_ = 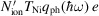. With an Ni mesh transmission of 80% and an ion-mean-charge of 7.6, this results in about 10^−18^ A, which is not sufficient for an XGMD measurement. The PETRA III line P01 at DESY offers from 2.5 keV up to 80 keV about 10^12^ photons s^−1^ monochromatic light. This corresponds at 29.3 keV radiation to about 10^5^ Xe ions s^−1^, which will lead to about 10^−13^ A, which is at the edge for XGMD measurements and would produce a huge uncertainty that, in turn, will limit future pulse energy evaluations. Additionally, we would need a bolometer or photodiode at a not absolutely calibrated Synchrotron beamline. An upgrade of the Elettra synchrotron facility is planned for 2027 to reach up to 10^13^ photons s^−1^ at photon energies up to 50 keV. To our knowledge, currently only the refurbished ID15A beamline of ESRF provides at 50 keV in the range of 10^13^ photons s^−1^ (Vaughan *et al.*, 2020 ▸). This increase by one order of magnitude allows for proper XGM calibration data at higher photon energies. Calibration of XGMDs directly at EuXFEL is also limited: they have to be measured together with a bolometer which can only be installed in an experimental hutch. Note that a bolometer measures fundamental photon energy including higher harmonics. The higher harmonics have a much lower cross-section and can usually be neglected in XGMD measurements. Thus, a method to attenuate the fundamental radiation and to transmit the harmonics has to be developed in order to measure the harmonic background on the bolometer. Ideally, a monochromator would be used for this purpose, but currently no monochromators above 24 keV are available at EuXFEL.

A straightforward and fast approach to extend the operation range above 25 keV is to extrapolate the photo-ionization cross-sections and ion-mean-charges for Kr and Xe. This is reasonable, since the cross-section as a function of photon energy has a quasi linear dependence on a log–log plot and the ion-mean-charge is almost constant at these photon energies (not shown). Additionally, we increased the uncertainty for the cross-sections and ion-mean-charges for the extrapolated data from typically 3–5% to 10% and from 0.5% to 2%, respectively, based on the typical variation of the measured data points.

**Figure 4:**
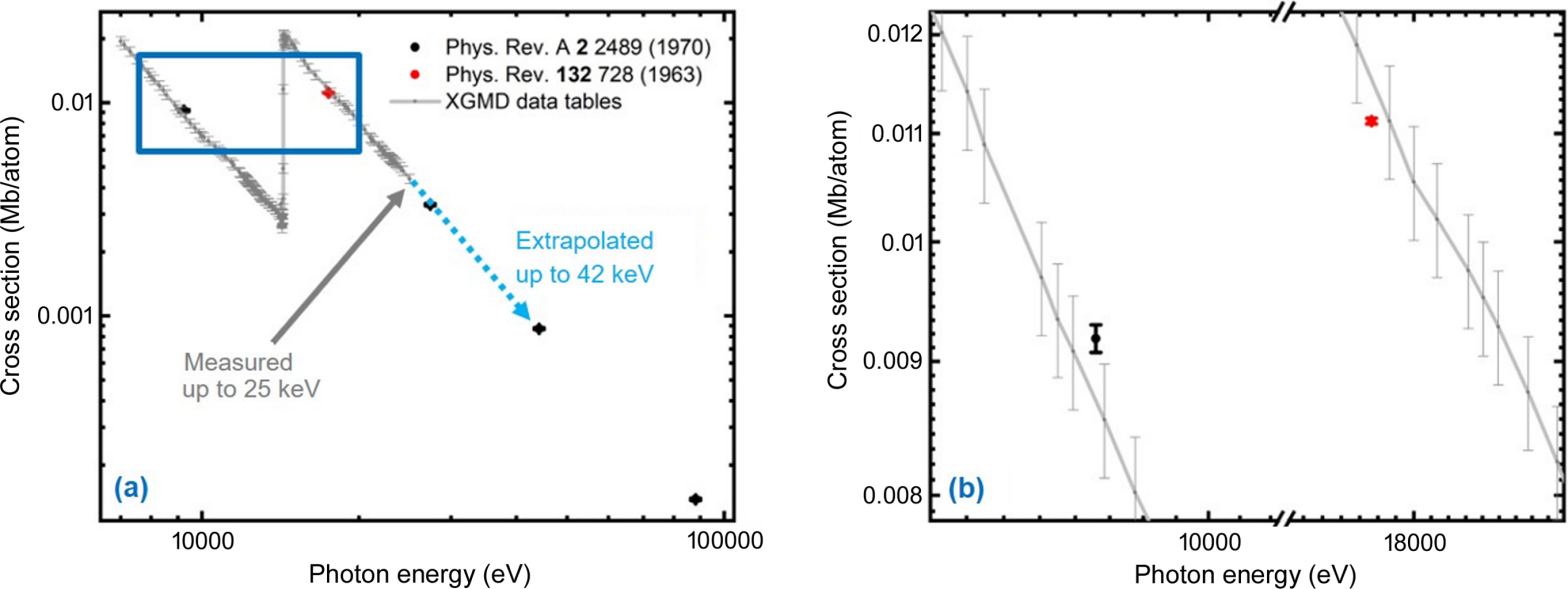
Cross-sections for Kr as a function of photon energy in a log–log plot: XGM values (gray) are photo-ionization only, whereas the literature values are total cross-sections. Extrapolation of XGM data up to 42 keV is indicated with a light-blue dotted line. (*b*) Enlargement of the area marked in (*a*).

For cross-sections, there is an overview paper from 100 eV up to 100 keV (Saloman *et al.*, 1988 ▸). From this the cross-sections for Kr (McCrary *et al.*, 1970 ▸; Chipman & Jennings, 1963 ▸) and Xe (McCrary *et al.*, 1970 ▸; White, 1934 ▸) were extracted. Note that the authors measured absorptions at single photon energies and derived the total cross-section, which includes the photo-ionization and scattering cross-section. For the XGMs, the photo-ionization cross-section is needed, but here all the available literature data were taken for comparison with the extrapolations. Figs. 4 ▸ and 5 ▸ show cross-sections for Kr and Xe, respectively. The literature values for single photon energies, which exist for even up to almost 100 keV, were plotted together with the XGM data tables (gray), which exist up to 25 keV. The extrapolation is indicated with a light blue dotted line. The Kr photo-ionization cross-sections were extrapolated up to 42 keV, whereas for Xe we stopped at 34.4 keV before the last absorption line (at 34.565 keV). In the case of Kr, the XGM data tables fit within the uncertainties of the literature values, although the literature values are total values and not just photo-ionization cross-sections. In the case of Xe, discrepancies are found outside the uncertainty [note White (1934 ▸) does not give any uncertainties]. Thus, we do not trust data above the last resonance and stopped the extrapolation at 34.4 keV. Since the XGM cross-section together with the ion-mean-charge tables always gave reliable and reproducible pulse energies, which always agreed with independent bolometer measurements, we decided to extrapolate the XGM data tables with increased uncertainty.

**Figure 6:**
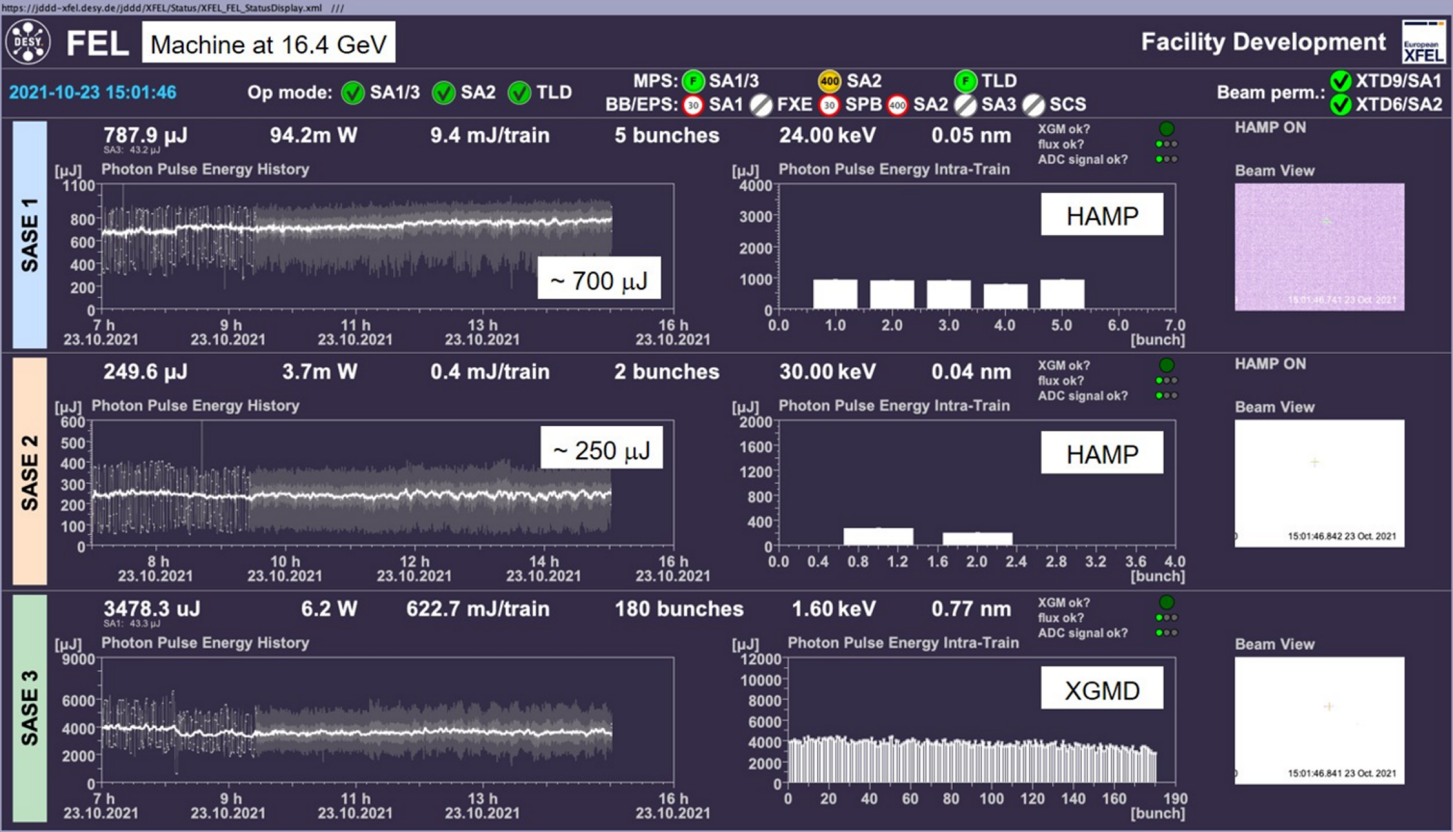
EuXFEL operation at very hard X-rays: single-shot upstream XGM detectors for SASE1 and SASE2 were HAMP (Xe gas target), whereas SASE3 was operated with XGMD (Kr gas target). The electron bunches were accelerated to 16.4 GeV. The SASE1, SASE2 and SASE3 photon energies were 24 keV, 30 keV and 1.6 keV, respectively. Left: graphs are the arithmetical averages over the single-shot pulse energies. Right: actual single-shot pulse energies plotted as bars. The average light powers at all SASE sources are shown.

These extrapolated XGM reference data were used during an EuXFEL accelerator run at 16.4 GeV. Fig. 6 ▸ shows a screenshot of SASE1, SASE2 and SASE3 operated at 24 keV, 30 keV and 1.6 keV, respectively. The SASE1 operation at 24 keV still uses the measured XGM tables for Xe, whereas the SASE2 run at 30 keV uses the extrapolated Xe cross-section and ion-mean-charge. The absolutely calibrated XGMD pulse energies had an uncertainty of 8% in SASE1 and 7% in SASE3, which are the common values. In SASE2 with 30 pulses per train (Fig. 6 ▸ shows 2 pulses per train), the XGMD uncertainty was 22%. It could have been slightly reduced if hundreds of pulses per train were used, which was technically not possible in that run. However, a higher uncertainty will always remain due to the extrapolation of the cross-sections and ion-mean-charges. HAMP was used as the single-shot detector for SASE1 and SASE2, whereas XGMD was used at 1.6 keV in SASE3. All single-shot signals were calibrated to the absolutely calibrated XGMD signal of the corresponding XGM.

For a proper XGM operation, only ionizing photons of one photon energy have to pass through the gas target. To block synchrotron radiation light at the EuXFEL upstream XGMs, graphite filters and synchrotron radiation apertures are used. For example, above 12 keV the graphite filter becomes necessary, because an aperture alone is not sufficient and the upstream XGM starts to overestimate the pulse energies due to the lower photon energy of the background that has a higher cross-section at the XGM gas target. At 30 keV, even with a graphite filter and closed apertures, a remaining influence of the background radiation was found. Closing the apertures from the usual 3–4 mm at 9–12 keV to 1.5 mm and even below helped in that case. The remaining background can be measured by suppressing the SASE lasing of the electron bunches, and thereafter it can be subtracted from further measurement data.

## 5. Estimation of XGM performance at 50 keV

In this section, the XGM sensitivity at 50 keV SASE operation will be estimated. Since there are no experimental data, we will assume a SASE operation with at least 10^9^ photons per pulse at 50 keV photon energy. Chen *et al.* (2021 ▸) calculated up to the maximal 0.16 mJ at 45 keV at full SASE optimization, which would be 2 × 10^10^ photons per pulse. Xe is used as the target gas, because after the last resonance it has the highest available cross-section and thus the highest possible signal. Additionally, a cross-section of 0.00257 Mb (= 2.57 × 10^−25^ m^2^) from the literature at 50 keV and an ion-mean-charge of 7.6 like before the last xenon resonance will be used. All these values are assumptions, but here only orders of magnitude will be discussed. If 10^9^ photons per pulse is used in equation (1) at 50 keV together with a temperature of 296 K, a detector length of 0.22 m and the maximum pressure of 1 × 10^−4^ mbar (= 1 × 10^−2^ Pa), this results in 138 ions per pulse. For the single-shot HAMP statistics, 138^1/2^/138 = 9% is much higher than the usual 1–2%, but this may still be acceptable for many applications. 138 ions per pulse at 50 keV with 1 pulse per train, 10 Hz train repetition rate and an Ni mesh transmission of 80% lead [using equation (2)] to an average photo-ion current of 1.3 × 10^−15^ A, which is far below the XGMD resolution. But at 100 pulses per train, which is easily achieved at EuXFEL, this would be 1.3 × 10^−13^ A, which is at the lower edge of XGMD sensitivity. Therefore, as photon energies become higher, the absolute calibrated integral XGMD signal performance becomes more dependent on the number of pulses per train and the single-shot pulse energies. The uncertainty of the absolutely calibrated average pulse energy at these high photon energies will be worse than the usual 7–10%, but we can not estimate it here because it depends on how precisely we can measure the ion-mean-charge and photo-ionization cross-sections at synchrotrons.

Note that further increasing the operating gas pressure above 1 × 10^−4^ mbar increases the risk of high-voltage sparking within the detectors. Additionally, at higher gas pressures the photo-electrons will also start to excite the gas atoms, which will lead to a new source of charges that is not directly related to the X-ray photon beam. Another way to increase the charge yield is to construct longer detectors and vacuum chambers, but since equation (1) is linear, we need a factor of ten longer detector if we want to gain a factor of ten in signal, which is technically very difficult. Another problem at higher photon energies could be that higher extraction voltages for photo-electrons and photo-ions are needed, which in turn could lead to sparking. An alternative method for single-shot pulse energy measurements are solid-state detectors. At EuXFEL, single-shot position measurements with a diamond detector have been demonstrated by Yıldız *et al.* (2023 ▸). In the future, these signals could be calibrated to the absolute integral XGMD signal or to a bolometer and could be used as an alternative for HAMP.

## 6. Summary and outlook

We have discussed the XGM operation at EuXFEL at high photon energies. We extrapolated the cross-sections and ion-mean-charges with an increased uncertainty to photon energies above 25 keV. Above 18 keV, HAMP is needed as single-shot detector, because the XGMD detector is far above the designed range and suffers from a diminishing SNR. We demonstrated reliable HAMP operation and a single-shot pulse energy correlation coefficient of around 0.98, which is as good as that achieved with the XGMD detector. An intra-train non-linearity at the HAMP detector was found but a signal level below 2 mV mitigates this effect. Additionally, the upper repetition rate limit for HAMP operation was found at 2.25 MHz since the single-shot peaks overlap at 4.5 MHz. Finally, we estimated the XGM performance at 50 keV and showed that it is still possible to measure the pulse energies with certain limitations: the single-shot uncertainty will increase from 1% to 9% and the average pulse energy measurement will be impossible for a single pulse/train, thus at least 100 pulses per train are needed to reach the XGMD lower detection limit.

## Figures and Tables

**Figure 5:**
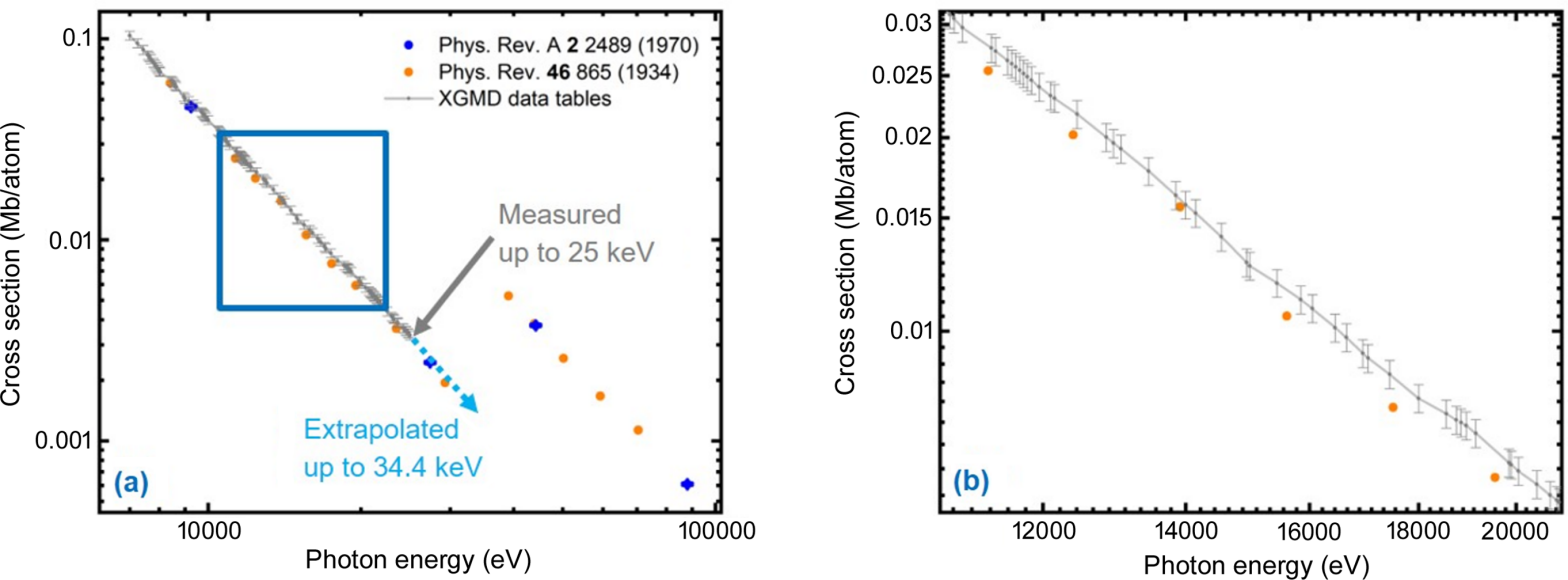
Cross-sections for Xe as a function of photon energy in a log–log plot: XGM values (gray) are photo-ionization only, whereas the literature values are total cross-sections. Extrapolation of XGM data up to 34.4 keV is indicated with a light-blue dotted line. (*b*) Enlargement of the area marked in (*a*).
